# Specific humoral and cellular immunity induced by *Trypanosoma cruzi* DNA immunization in a canine model

**DOI:** 10.1186/1297-9716-44-15

**Published:** 2013-03-11

**Authors:** Minerva Arce-Fonseca, Martha A Ballinas-Verdugo, Emma R Abreu Zenteno, Davinia Suárez-Flores, Silvia C Carrillo-Sánchez, Ricardo Alejandre-Aguilar, José Luis Rosales-Encina, Pedro A Reyes, Olivia Rodríguez-Morales

**Affiliations:** 1 Department of Molecular Biology, Instituto Nacional de Cardiología “Ignacio Chávez”, Juan Badiano No. 1, Col. Sección XVI, Tlalpan, Mexico City, CP 14080, Mexico; 2 Department of Parasitology, Escuela Nacional de Ciencias Biológicas, Instituto Politécnico Nacional, Prolongación de Carpio y Plan de Ayala, Col. Santo Tomás, Miguel Hidalgo, Mexico City, CP 11340, Mexico; 3 Department of Infectomics and Molecular Pathogenesis, Centro de Investigación y de Estudios Avanzados-IPN, Av. Instituto Politécnico Nacional No. 2508, Col. San Pedro Zacatenco, Gustavo A. Madero, Mexico City, CP 07360, Mexico; 4 Research Direction, Instituto Nacional de Cardiología “Ignacio Chávez”, Juan Badiano No. 1, Col. Sección XVI, Tlalpan, Mexico City, CP 14080, Mexico

## Abstract

Chagas disease has a high incidence in Mexico and other Latin American countries. Because one of the most important known methods of prevention is vector control, which has been effective only in certain areas of South America, the development of a vaccine to protect people at risk has been proposed. In this study, we assessed the cellular and humoral immune response generated following immunization with pBCSP and pBCSSP4 plasmids containing the genes encoding a trans-sialidase protein (present in all three forms of *T. cruzi*) and an amastigote specific glycoprotein, respectively, in a canine model. Thirty-five beagle dogs were divided randomly into 5 groups (*n* = 7) and were immunized twice intramuscularly with 500 μg of pBCSSP4, pBCSP, pBk-CMV (empty plasmid) or saline solution. Fifteen days after the last immunization the 4 groups were infected intraperitoneally with 500 000 metacyclic trypomastigotes. The fifth group was unimmunized/infected. The parasitaemia in the immunized/infected dogs was for a shorter period (14 vs. 29 days) and the parasite load was lower. The concentration of IgG1 (0.612 ± 0.019 O.D.) and IgG2 (1.167 ± 0.097 O.D.) subclasses was measured (absorbance) 15 days after the last immunization with both recombinant plasmids, the majority of which were IgG2. The treatment of parasites using the serum from dogs immunized with pBCSP and pBCSSP4 plasmids produced 54% (± 11.8) and 68% (± 21.4) complement-mediated lysis, respectively. At 12 h post immunization, an increase in cytokines was not observed; however, vaccination with pBCSSP4 significantly increased the levels of IFN-γ and IL-10 at 9 months post-infection. The recombinant plasmid immunization stimulated the spleen cell proliferation showing a positive stimulatory index above 2.0. In conclusion, immunization using both genes effectively induces a humoral and cellular immune response.

## Introduction

Chagas’ disease, caused by the parasite protozoan *Trypanosoma cruzi,* is estimated to affect 10 million people in the Americas [1]. To date, the most important prevention method is to campaign for the elimination of the vector; however, these efforts have achieved only partial control of the disease. There is a need for an immunoprotective vaccine to decrease the morbidity and mortality caused by this parasitic disease. Many attempts have been made to generate a vaccine using the *T. cruzi* highly immunogenic surface antigens, recombinant antigens, and recently, the administration of test plasmid DNA encoding different genes has been investigated. The results of these tests have varied from no protection against the disease to the partial reduction of short-term mortality and morbidity rates in a murine model [2-4]. Although there have been a number of successes using this type of vaccine, it is necessary to improve the vaccine-mediated immune response, to document the results in detail, and to replicate the experiments in other species. The canine model is ideal because dogs develop the signs of the disease and immunopathological changes that are similar in humans; in addition, dogs are considered important domestic reservoirs of the parasite and contribute to the transmission of *T. cruzi* to humans; therefore, a decrease in the incidence of the disease in this species would have significant beneficial effects for humans [5-7].

In the case of Chagas disease, the cell-type immune response plays a fundamental role because the parasite exhibits an intracellular phase during the infection. One of the best indicators of the establishment of a cellular immune response is the production of type 1 cytokines, whereas type 2 cytokines promote the antibody response.

It has been established that the predominance of Th1 cytokines is more effective in eliminating the parasite and therefore, reduces parasitaemia during the acute phase. In the later stages of the disease, Th1/Th2 cytokines are associated with the absence of symptoms and the apparent integrity of the cardiac tissue [8,9]. Both cellular and humoral immune responses are driven by CD4 + T lymphocytes (Th1 and Th2) through signals generated by cytokines. In a previous study, a cDNA clone encoding the amastigote-specific surface protein *Tc*SSP4 was used as a candidate gene to develop a DNA vaccine. The mice were immunized with the recombinant protein *rTc*SSP4 and with the *TcSSP4* cDNA and were challenged with the introduction of blood trypomastigotes. The immunization with the *rTc*SSP4 protein rendered the mice more susceptible to trypomastigote infection and exhibited high mortality rates, whereas the mice immunized with a eukaryotic expression plasmid containing the *TcSSP4* cDNA controlled the acute phase of the infection. Compared with the control animals, the heart tissue of the DNA-vaccinated animals did not demonstrate myocarditis and tissue damage at 365 days following infection. Interferon-gamma (IFN-γ) was detected in the sera of the DNA-vaccinated mice shortly after immunization, suggesting the development of a Th1 response. Therefore, the *TcSSP4* gene is a promising candidate for the development of an anti-*T. cruzi* DNA vaccine [10].

In this study, the humoral and cellular immune responses were evaluated in dogs immunized with 2 *T. cruzi* genes, *TcSP* (gene encoding a member of the trans-sialidase family that is present in all three forms of *T. cruzi*) [11] and *TcSSP4*, through the measurement of antibodies and cytokines in the serum, antibody-dependent cell lysis, and the in vitro proliferation of the dog splenocytes.

## Materials and methods

### Animals

Male and female 4-month-old Beagle dogs (35 in total) were purchased from Criadero El Atorón (Teotihuacán, Estado de México; Mexico). All animals were subjected to a physical examination and were quarantined. The dogs were dewormed and vaccinated against the main canine infectious diseases. The dogs were tested for the absence of antibodies against *T. cruzi* using the (ELISA). The animals were handled in accordance with the guidelines established by international authorities and the Norma Oficial Mexicana technical specifications for the care and use of laboratory animals [12]. The Bioethics Committee of the Instituto Nacional de Cardiología, Ignacio Chávez approved the experimental protocol.

### Immunization and challenge

The dogs were divided randomly into 5 groups (*n* = 7). The groups were immunized with pBCSSP4, pBCSP, pBk-CMV (empty plasmid) or saline solution (SS) (used as a control), and 15 days after the last immunization, the 4 groups were infected with the parasite. The two plasmid constructs (pBCSSP4 and pBCSP) were generated and characterized as described previously [11]. The immunizations consisted of 2 intramuscular injections of 500 μg of DNA dissolved in 500 μL of SS administered at 2-week intervals.

The parasites were obtained from urine and feces of triatomes and resuspended in physiologic solution. The animals in the fifth group were administered 500 000 metacyclic trypomastigotes intraperitoneally. All the animals survived the treatment and they are currently being used in other studies. The challenge was performed using the Ninoa strain of *T. cruzi* that was isolated from a patient in Mexico [13] and was maintained by serial passages in triatomines.

### Parasitaemia

The parasitaemia was determined microscopically by examining freshly isolated blood samples collected from the brachiocephalic vein of the infected animals every third day, starting on day 10 post-infection until day 65 post-infection.

### *T. cruzi* anti-IgG determination using enzyme-linked immunosorbent assay (ELISA)

A standardized ELISA to detect the anti *T. cruzi* antibody using as the antigen a total extraction of *T. cruzi* INC-9 isolate of epimastigote forms [14] was performed to rule out any previous natural infections in all dogs, to establish the production of antibodies following immunization in immunized/non-infected dogs, and to confirm the experimental infection two months after challenge in all infected/unimmunized dogs.

### Indirect immunofluorescence (IIF) assay

The ELISA-reactive sera were processed by IIF using the method described as follows: INC-9 *T. cruzi* epimastigote suspension was placed on a slide and fixed. A 1:40 dilution of the test serum was made in 1X phosphate buffered saline (PBS), incubated in a humidified chamber at room temperature for 1 h, and washed under stirring in PBS three times (5 min for each wash). The slides were covered with an optimized dilution (1:100) of fluorescein isothiocyanate- labeled goat anti-dog IgG conjugate (Novus Biologicals). The slides were examined with a UV epifluorescence microscope at 3200 and 3400 magnification for specific trypanosomal fluorescence. Positive fluorescence was defined as detection of green fluorescence on parasites and was labeled with + /+ + + + according to the fluorescence degree watched, using a positive control as the reference point [14].

### Complement-mediated lysis assay

The metacyclic trypomastigotes were obtained from the urine and feces of triatomines and were resuspended in SS; 2 × 10^4^ parasites were incubated with 5 μL healthy dog sera or test serum (immunized/non-infected dogs) for 1 h at 37°C. Guinea pig complement (25 μL) (Faculty of Veterinary Medicine, Universidad Nacional Autónoma de México) was added, and the tubes were incubated for 2 h at 37°C. The control tubes contained parasites incubated with test serum and heat-inactivated complement (56°C for 30 min), and parasites incubated with pre immune serum and complement. The samples were assayed in triplicate, and the number of motile trypomastigotes was determined using a hemocytometer. The percentage of antibody-dependent cell lysis was calculated by determining the percent killing = 100 – [(number of parasites after incubation with guinea pig complement/number of parasites after treatment with heat-inactivated complement) × 100], as previously reported [15].

### Cytokine analysis

The cytokine levels in the sera of immunized dogs at 12 and 24 h after the last immunization, or 9 months after the infection, were measured using ELISA. The ELISA for IFN-γ, interleukin-10 (IL-10) and tumor necrosis factor-alpha (TNF-α) were performed in accordance with the manufacturer’s instructions (Preprotech, Rocky Hill, NJ, USA).

### Cell proliferation assay

On day 15 after the last DNA immunization or SS inoculation, and before the challenge, a partial splenectomy was performed [16]. The canine spleen cells were washed 3 times in Hank’s solution (Sigma Aldrich, St. Louis, MO, USA) and were resuspended in Dulbecco’s Modified Eagle Medium (DMEM, Gibco BRL) supplemented with 2 mM L-glutamine, 50 μM β-Mercaptoethanol, 1% (vol/vol) non-essential amino acid solution and 10% fetal bovine serum, to a concentration of 4 × 10^5^ cells/mL. The assay was performed in 96-well flat bottom plates (Corning); 100 μL of the cell suspension was placed into each well, and the total parasite extract of epimastigote forms was added to a concentration of 10 μg/mL. Concanavalin A (Con A) (Sigma Aldrich, St. Louis, MO, USA) was added to a concentration of 2 μg/mL as a positive control. Each determination was performed in duplicate. The cultures were incubated at 37°C in 5% CO_2_ for 120 h (or 72 h for Con A). At 16 h prior to the end of the incubation, 0.5 μCi of [^3^H]-thymidine (Amersham) were added to each well. The lymphocytes were collected using a manual cell harvester, and the amount of incorporated radioactive thymidine was measured using liquid scintillation spectroscopy (Beckman, LS 5801). The stimulation of a specific cellular immune response is represented by the stimulatory index (S.I.) [17].

### Statistical analysis

All the data were analyzed using one-way ANOVA statistics followed by Tukey’s analysis. In all cases, differences were considered significant when *P* < 0.05.

## Results

### Parasitaemia and establishment of infection

All of the experimental animals tested negative in the ELISA prior to the start of the experiment.

All unimmunized/infected dogs exhibited parasitaemia starting on day 22 and lasting until day 51 post-infection, whereas the parasitaemia in the immunized/infected dogs was for a shorter period, from day 32 to 46 post-infection (Table 1). Parasitaemia was too low to be quantified, and parasites were only seen by direct observation. The limit of detection was 200 to 400 parasites/mL of blood sample intermittently throughout all analysis. At two months post-inoculation, the *T. cruzi* infection was diagnosed by the ELISA method and was confirmed by IIF in all unimmunized/infected dogs (data not shown). All animals survived the infection with the parasite, because the strain used is not lethal. However, these dogs showed cardiopathy demonstrated in electrocardiographic studies such as left ventricle enlargement, intraventricular conduction defects, right bundle branch block and ventricular premature complexes. In immunized/infected dogs, the quality and quantity of the electrocardiographic abnormalities diminished [11].

**Table 1:** **Parasitemia detection in DNA-immunized dogs with experimental *T. cruzi *infection**

**Group**	**Dog**	**Days after inoculation**									
		**10-15**	**16-20**	**21-25**	**26-30**	**31-35**	**36-40**	**41-45**	**46-50**	**51-60**	**6165**
pBCSP	1							+	+		
	2					+					
	3										
	4										
	5										
	6										
pBCSSP4	1						+				
	2						+				
	3										
	4										
	5										
	6										
pBk-CMV	1					+	+				
	2					+	+	+			
	3					+	+				
	4										
	5										
	6										
SS mock-immunized	1						+ +	+ +		+	
	2						+ +				
	3						+ +	+ +	+	+	
	4					+				+	
	5			+ +	+ +		+ +				
	6			+	+ +		+ +				

+ Detection of 1 or 2 parasites per blood sample of fresh drop examination.

### Humoral immune response

**Figure 1:**
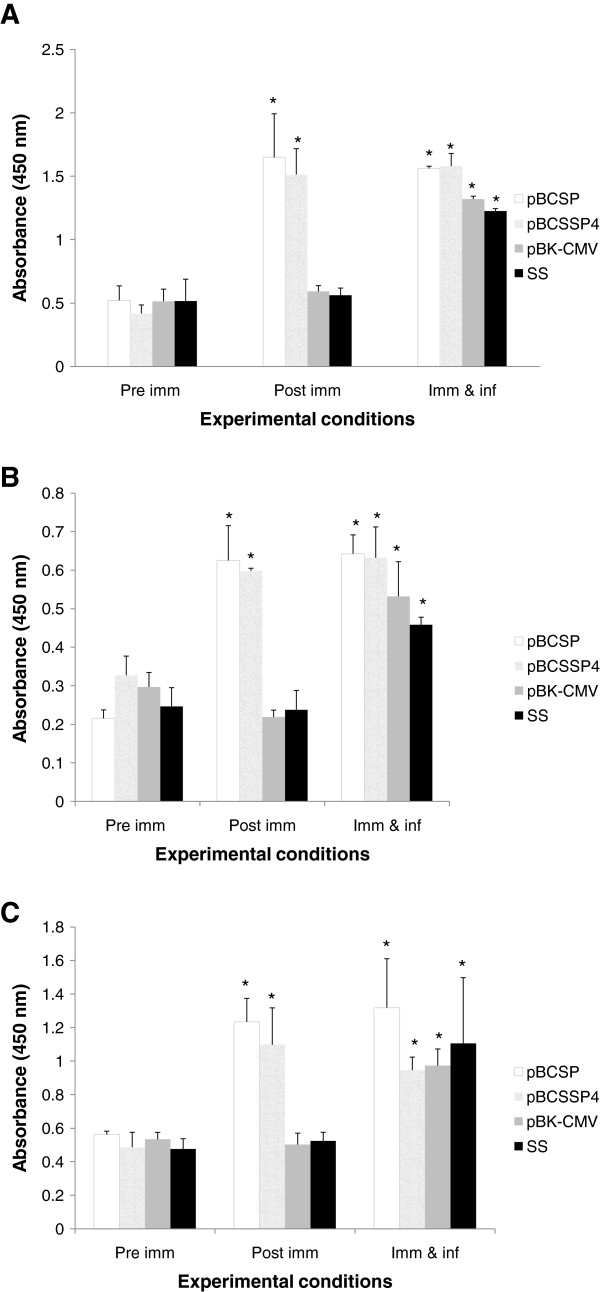
**The antibody response to *T. cruzi *infection in immunized dogs. **An ELISA was performed to evaluate the serum levels (absorbance in optical density at 405 nm) of *T. cruzi*-specific antibodies at different times. (**A**) IgG, (**B**) IgG1, and (**C**) IgG2. To determine all immunoglubulins, 1 μg/μL of total *T. cruzi *extract was used, and the anti-dog IgG, IgG1 and IgG2 antibodies (Novus Biologicals) were diluted 1: 10 000 (IgG), 1:5000 (IgG1) and 1:30 000 (IgG2) in blocking buffer. Pre imm: before the immunization, Post imm: 15 days after the last immunization, and Imm & inf: 60 days after the infection in immunized dogs. The values represent the average of triplicate assays ± S.D (* *P *< 0.05).

Determination of the parasite-specific immunoglobulin G (IgG) antibodies using ELISA revealed that immunization with both recombinant plasmids induced a significant production of IgG against the parasite 15 days after the last immunization (immunized/non-infected dog determination of the parasite-specific immunoglobulin G (IgG) antibodies using ELISA revealed that immunization with both recombinant plasmids induced a significant production of IgG against the parasite 15 days after the last immunization (immunized/non-infected dogs). As shown in Figure 1B, ELISA analysis shows that recombinant plasmid immunized/non-infected dogs displayed *T. cruzi*-specific IgG1 subclass levels significantly higher with respect to SS-inoculated/non-infected and pBk-CMV-immunized/non- infected dogs.

The DNA immunization with recombinant plasmids induced a predominant Th1 type antibody response because of the IgG produced, the majority belonging to the IgG2 subclass and to a lesser extent, to the IgG1 subclass (Figure 1C).

As expected, all dogs exhibited high levels of antibodies against *T. cruzi* after challenge (Figure 1).

### Complement-mediated lysis

To assess the biological activities of the antibodies induced by the DNA immunization, we performed complement-mediated parasite lysis assays. The sera of dogs immunized with the recombinant plasmids were in contact with the metacyclic trypomastigotes and guinea pig complement. The antibodies produced by the dogs immunized with the recombinant plasmids induced the complement-mediated lysis of trypomastigotes in vitro. The specific lysis of parasites was not detected when the pre immune serum or the serum from dogs immunized with the control empty plasmid or SS were used.

**Figure 2:**
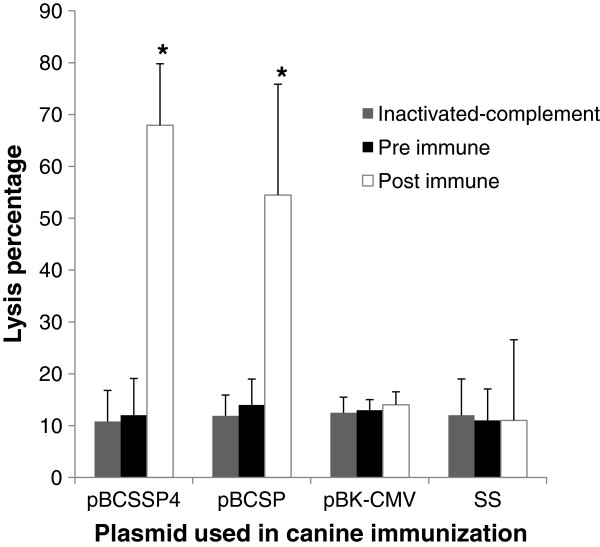
**Percentage of complement-mediated lysis of trypomastigotes. **Percent killing = 100 – [(number of parasites after incubation with guinea pig complement/number of parasites after treatment with heat-inactivated complement) × 100]. Inactivated-complement: test serum (post immune obtained 15 days after the last immunization) and heat-inactivated complement (56°C for 30 min), Pre immune: sample obtained before the immunization and complement, and Post immune: test serum (obtained 15 days after the last immunization) and complement. All dogs were non-infected. The values represent the average of triplicate assays ± S.D (* *P* < 0.05).

Treating the parasites with the serum from the dogs immunized with pBCSSP4 or pBCSP plasmids resulted in 68% (± 21.4) and 54% (± 11.8) lysis, respectively (Figure 2); the results with both plasmids were similar.

### Cytokine quantification

**Figure 3:**
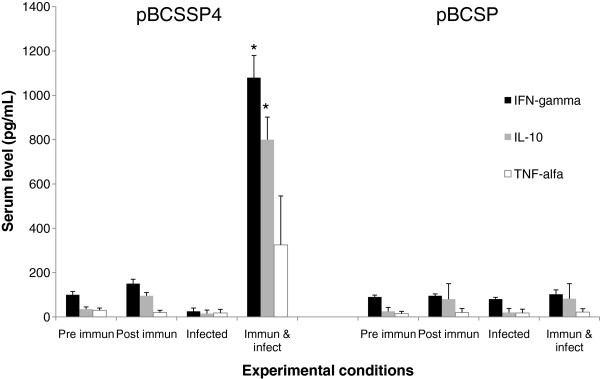
**Cytokine response in pBCSSP4- and pBCSP-immunized dogs. **An ELISA was performed to evaluate the serum levels of IFN-γ, IL-10, and TNF-α at different times. Pre immune: before the immunization, Post immun: 15 days after the last immunization, Infected: 9 months post infection, and Immun & infect: 9 months post infection in immunized dogs. The values represent the average of triplicate assays ± S.D (* *P *< 0.05).

The DNA vaccines tested did not generate increased cytokines at 12 h post immunization; however, at 9 months post-infection, although both DNA vaccines were tested, only the pBCSSP4 plasmid induced a significant increase in IFN-γ (1080 pg/mL ± 100) and IL-10 (800 pg/mL ± 102) levels in the dogs (Figure 3) at this same time, the values obtained with the pBCSP plasmid-immunization for IFN-γ (100 pg/mL ± 20), IL-10 (102 pg/mL ± 68), and TNF-α (221 pg/mL ± 15) were similar to those observed at the different experimental conditions analyzed (Figure 3).

### Lymphoproliferative response

To evaluate the specific cellular immune response in the dogs immunized intramuscularly with the genes from *T. cruzi* (the dogs were not infected), we studied the lymphoproliferative response to the parasite antigens.

Con A was used as a positive control; since in the presence of a nonspecific mitogen, the cells necessarily proliferate if they are viable.

**Figure 4:**
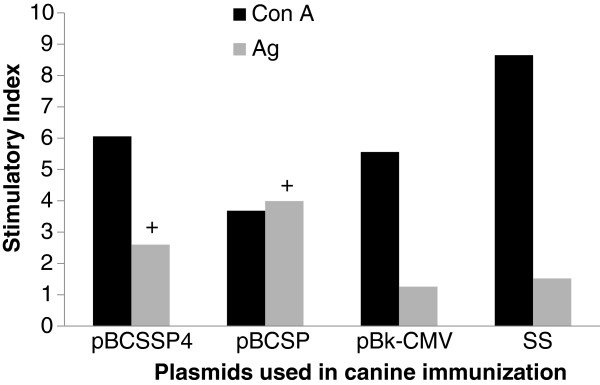
**Spleen lymphocyte proliferation in immunized dogs. **The values represent the S.I. estimated as follows: mean counts per minute of stimulated cultures/mean counts per minute of non-stimulated cultures. S.I. values equal to or above 2.0 were considered positive (+). Con A: concanavalin A and Ag: parasite total protein extract of epimastigote forms.

Positive lymphoproliferative responses were observed in the animals immunized with the *T. cruzi* genes. The highest lymphocyte S.I. (3.99) was observed in the dogs immunized with the pBCSP plasmid, and although there was less proliferation in the splenocytes from the dogs immunized with pBCSSP4, the S.I. (2.6) was positive (Figure 4), showing a value above 2.0 when the lymphocyte cultures were stimulated with the parasite antigens. In the other groups (controls) there was no cell proliferation; the S. I. values were below 2.0. The cultures stimulated with Con A showed a mean S. I. value of 5.99 ± 2.05 demonstrating the cell viability in all experimental groups. These results suggest that the administration of nucleic acids from *T. cruzi* induces the specific stimulation of the cellular immune response.

## Discussion

In animal models, it has been shown that DNA immunization induces and modulates the immune response necessary for the prevention of infectious diseases [18], for the treatment of neoplastic disorders [19] and for allergic treatment [20] and autoimmune diseases [21]. Therefore, we employed this method of immunization to induce a specific immune response against *T. cruzi* in Beagle dogs.

Antibodies may contribute to the immunity against *T. cruzi* through complement-dependent cytolysis, opsonic and/or cytophilic antibodies that enhance phagocytosis in antibody-dependent cellular lysis reactions against the parasite, and possibly by interfering with the physiologic processes of the parasite. Generally, polyclonal B cell activation that leads to hypergammaglobulinemia and a delayed specific humoral immune response is accepted as a characteristic of acute phase Chagas disease in humans and is reported in rodent experimental models of *T. cruzi* infection [22,23]. Different IgG isotypes have been implicated in polyclonal B cell activation and parasitic-specific antibody responses; the major antibody isotypes involved in the elimination of parasite blood forms and in decreasing mortality rates are IgG1 and IgG2 [24,25].

The analysis of the IgG subclasses produced by the immunization of canines with both recombinant plasmids revealed that the dominant antibody produced was IgG2; in a previous study in mice immunized with the pBCSP plasmid, the dominant subclass was also IgG2a [26]. These results are consistent with other studies using DNA vaccines; for example, the majority of antibodies produced were of the IgG2 subclass following immunization of dogs with a multi-component DNA vaccine containing *T. cruzi* trans-sialidase family genes [27]. In addition, in a study performed by Pereira-Chioccola et al., mice were immunized with a plasmid containing the trans-sialidase gene, and the majority of the antibodies produced were of the IgG2a subclass, whereas following immunization with the recombinant protein, the dominant antibody subclass was IgG1 [28]. Because the subclass profile of antibodies reflects the type of activated helper T cells [29], the predominant IgG2 subclass production after plasmid vaccination with both genes in this study suggests a Th1 skewed response.

Various antibodies against *Trypanosoma cruzi* elicited by an active infection may have different functional activities. Antibodies that bind to live trypomastigote forms and lyse them in conjunction with complement in an in vitro reaction have been linked to active infection. These lytic antibodies, detected by the complement-mediated lysis test, are produced after infection, and have been used to evaluate treatment efficacy in Chagas’ disease in which the negative result of the complement-mediated lysis test has been interpreted as the absence of parasitemia and a cure. The Zulantay et al. results support the idea of the role of lytic antibodies in the control of parasitemia [30]. Lytic antibodies are not easy to elicit by immunization with dead parasite or with purified *T. cruzi* antigens, although they are readily detected in mice, rats, rabbits and humans infected with the parasite [31]. The present study indicates that immunization with pBCSP and pBCSSP4 plasmids induced the humoral immune response and that such antibodies mediated the lysis of parasites through the complement system. It has been previously reported that complement-mediated lytic antibodies are associated with protection. Our data are comparable to results reported by Sepúlveda et al. showing that the immunization of mice with DNA encoding a regulatory protein of the complement pathway of *T. cruzi*, protected against lethal challenge in a process that involved lytic antibodies [15]. It has been shown that the susceptibility of *T. cruzi* to lysis by complement varies according to the strain [32]; complement lysis assays showed that the Colombian *T. cruzi* strain is more susceptible to lysis than the Y *T. cruzi* strain at all serum concentrations [33]. Indeed, even within the same strain, variations in susceptibility have been observed among different clones [34]. There are no reports of *T. cruzi* lysis by complement with Ninoa strain; in our study the results showed a high percentage of lysis, similar to other Mexican *Trypanosoma cruzi* isolates reported by other authors [32].

The vaccination regimen used in our experimental model induced a cell-mediated immunity characterized by lymphoproliferation, as well as the enhanced production of IFN-γ, which was in agreement with the results from Cazorla et al. who studied mice immunized with a *T. cruzi* recombinant cruzipain (rCz); they measured the abundance of rCz-specific IFN-γ secreting- CD8+ T-cells in splenocytes depleted of CD4+ lymphocytes, and survival, efficient control of parasite load and restricted inflammatory myopathy were demonstrated [35]. Therefore, our experimental immunization appears promising for the development of a vaccine that generates similar results, since one of our antigens (TcSP) is an enzyme that is expressed in all developmental forms of the parasite (epimastigote, amastigote and trypomastigote) and it is also present at the surface level, characteristics shared with cruzipain.

Previously, immunization with the pBCSSP4 plasmid in a murine model induced significant amounts of IFN-γ at 3 hours post immunization, and significant amounts of IL-6 and TNF-α at 3 and 12 h post immunization [10]; however, in our canine model, immunization with pBCSSP4 or pBCSP did not induce cytokine production at 12 h post immunization. At 9 months after infection with *T. cruzi* in the dogs previously vaccinated with the pBCSSP4 plasmid, high levels of IFN-γ (1080 pg / mL) were observed and TNF-α was present to a lesser extent. These results together with the high production of IgG2a subclass indicate a Th1 pattern, and were consistent with reports from Aparicio-Burgos et al.; the circulatory cytokine levels (IFN-γ and IL-10) were below the detection limits before and after immunization with a multi-component DNA-prime/DNA-boost vaccine TcVac1— vaccine constituted of antigen-encoding plasmids (pCDNA3.TcG1, pCDNA3.TcG2 and pCDNA3.TcG4) and IL-12- and GMCSF-expression plasmids—, and all the dogs responded to infection with increases in IFN-γ levels (580 pg/mL approximately at 15 days post infection) [27].

During the testing of a therapeutic DNA vaccine encoding the TSA-1 antigen of *T. cruzi* in a murine model, the immunized mice exhibited significant increases in CD4 and CD8 T cells producing IFN-γ during the chronic phase of infection. This increase in IFN-γ production was associated with reduced inflammation of the cardiac tissue and a reduction in the parasite load [36]. Moreover, immunization with pELI_Tc2 (DNA from *T. cruzi*) produced elevated levels of IFN-γ that were associated with lower parasite loads after challenge [37]. In accordance with these results, the present study demonstrates that the recombinant plasmid vaccination induced high levels of IFN-γ, shorter parasitaemia, lower parasite load and also cardiac and clinical protection in immunized/infected dogs, the latter recently reported by Rodríguez-Morales et al. [11].

In dogs at the late stage of infection with *T. cruzi* characterized by the absence of heart damage, high levels of IL-10 in the serum and supernatants of peripheral blood mononuclear cells have been observed [7]. The significant increase in IFN-γ and IL-10 in the group immunized with the pBCSSP4 plasmid is attributed to the *TcSSP4* gene, or to the combination of this gene and the presence of the parasite (or the parasite antigenic molecules). The increase in IFN-γ and IL-10 cannot be due to the infection alone because an increase in the synthesis of the cytokines was not observed in the other infected groups. Therefore, immunization modulates the pattern of cytokine expression in infected dogs.

The results presented in this study support DNA immunization as a strategy for designing anti-*T. cruzi* vaccines. It was demonstrated that this type of vaccine effectively induces antigen-specific antibodies and produces a Th1-type cellular immune response reflected in a moderate cardiac and clinical protection against Chagas disease because this strategy 1) avoided acute phase fever, 2) induced an immune response that manifested as lymph node enlargement as part of host-protective activity, 3) avoided heart rate increases during the acute and/or chronic stages, and 4) most interestingly, halted the symptomatic progression to severe heart conduction abnormalities [11].

These vaccines will allow us to determine if this immune response confers protection against acute and chronic *T. cruzi* infection, the course of the infection, the pathological variables, and the clinical status in Beagle dogs with experimentally induced trypanosomiasis*.*

## Abbreviations

O.D: Optical density; rTcSSP4: Recombinant protein *Tc*SSP4; TcSSP4: Gene encoding an amastigote-specific surface protein in *T. cruzi*; IFN-γ: Interferon-gamma; TcSP: Gene encoding a member of the trans-sialidase family in *T. cruzi*; pBk-CMV: Commercial empty plasmid used as a cloning vector; pBCSP: Plasmid containing the *TcSP* gene; pBCSSP4: Plasmid containing the *TcSSP4* gene; ELISA: Enzyme-linked immunosorbent assay; SS: Saline solution; IIF: Indirect immunofluorescence; PBS: Phosphate buffered saline; IL-10: Interleukin-10; TNF-α: Tumor necrosis factor-alpha; DMEM: Dulbecco’s Modified Eagle Medium; Con A: Concanavalin A; S.I: Stimulatory index; ANOVA: Analysis of variance.

## Competing interests

The authors declare that they have no competing interests.

## Authors’ contributions

MAF and ORM conceived the study, conducted the animal trials, collected and analyzed data, and completed manuscript preparation. MABV, ERAZ and DSF carried out the immunological studies. SCCS carried out the molecular studies. RAA, JLRE and PAR contributed to the concept and design of the study. All authors read and approved the manuscript.
